# Convergent RANK- and c-Met-Mediated Signaling Components Predict Survival of Patients with Prostate Cancer: An Interracial Comparative Study

**DOI:** 10.1371/journal.pone.0073081

**Published:** 2013-09-16

**Authors:** Peizhen Hu, Leland W. K. Chung, Dror Berel, Henry F. Frierson, Hua Yang, Chunyan Liu, Ruoxiang Wang, Qinlong Li, Andre Rogatko, Haiyen E. Zhau

**Affiliations:** 1 Uro-Oncology Research Program, Department of Medicine, Cedars-Sinai Medical Center, Los Angeles, California, United States of America; 2 Biostatistics and Bioinformatics, Department of Medicine, Cedars-Sinai Medical Center, Los Angeles, California, United States of America; 3 Department of Surgery, Cedars-Sinai Medical Center, Los Angeles, California, United States of America; 4 Department of Pathology, University of Virginia, Charlottesville, Virginia, United States of America; 5 Department of Pathology, Jilin University, Changchun, Jilin, China; University of Kentucky College of Medicine, United States of America

## Abstract

We reported (*PLoS One* 6 (12):e28670, 2011) that the activation of c-Met signaling in RANKL-overexpressing bone metastatic LNCaP cell and xenograft models increased expression of RANK, RANKL, c-Met, and phosphorylated c-Met, and mediated downstream signaling. We confirmed the significance of the RANK-mediated signaling network in castration resistant clinical human prostate cancer (PC) tissues. In this report, we used a multispectral quantum dot labeling technique to label six RANK and c-Met convergent signaling pathway mediators simultaneously in formalin fixed paraffin embedded (FFPE) tissue specimens, quantify the intensity of each expression at the sub-cellular level, and investigated their potential utility as predictors of patient survival in Caucasian-American, African-American and Chinese men. We found that RANKL and neuropilin-1 (NRP-1) expression predicts survival of Caucasian-Americans with PC. A Gleason score ≥8 combined with nuclear p-c-Met expression predicts survival in African-American PC patients. Neuropilin-1, p-NF-κB p65 and VEGF are predictors for the overall survival of Chinese men with PC. These results collectively support interracial differences in cell signaling networks that can predict the survival of PC patients.

## Introduction

After the implementation of prostate-specific antigen (PSA) screening, prostate cancer (PC) diagnosis became much more common. Since one of every 8–10 men diagnosed with PC dies of this disease, it is important to develop effective predictors to select those who need to be treated and avoid unnecessary treatment [1], [2]. Over the past decades, many predictive biomarkers, either associated with tissues or in biologic fluids, have been used to differentiate indolent from aggressive forms of PC. These markers are categorized broadly as tumor suppressors, oncogenes, transcription factors, and regulators of cellular metabolism, and phenotypes such as cell proliferation, apoptosis, invasion, migration and metastasis [2], [3]. We combined cell culture models with lineage relationship, *i.e.*, which share the same genetic background but differ in their aggressiveness, with animal models that display variability in their intrinsic invasiveness and metastatic potential to develop relevant cell signaling pathways closely mimicking the phenotypes and behaviors of clinical human PC. We conducted a comparative study using clinical PC tissues associated with known patient survival to test the hypothesis that the expression of certain cell signaling network biomarkers found in animal models driving PC cells to develop lethal bone and soft tissue metastases might be used as biomarkers to predict the progression and survival of PC patients. To seek a better understanding of potential interracial differences of cell signaling networks, we tested the hypothesis that different RANK- and c-Met-mediated downstream cell signaling components may predict the survival of prostate cancer patients with different racial backgrounds.

We previously reported that lethal PC progression to bone and soft tissue metastases is determined by the osteomimetic property of PC cells [4], [5]. We found that soluble factors such as β2-microglobulin (β2-M) and receptor activator of NF-κB ligand (RANKL) can drive PC and other human cancer cells to undergo epithelial-to-mesenchymal transition (EMT) and confer aggressive phenotypes including local invasion and distant metastases [6]–[12]. Among the cell signaling pathways we have studied the activation of RANKL-RANK signaling was of particular interest because this signaling pathway was activated in both animal models and clinical PC specimens [8], [12], and targeting RANKL with an anti-RANKL antibody, denosumab, has been highly effective in blocking the lytic bone lesions associated with men treated with androgen deprivation therapy [13]. RANKL-RANK signaling also was found to be involved in the expansion of the stem cell niche during the development of hormone-sensitive organs [14], [15]. We observed in both LNCaP and ARCaP cell and animal models that a “vicious cycle” of RANKL-RANK signaling is responsible for conferring the ability of these cells to grow and metastasize to bone and soft tissues in mice, through the induction of EMT, local invasion and distant metastases [5], [8], [12]. By genetically inactivating RANK or c-Met receptor, we completely abrogated the ability of these cancer cells to metastasize to bone and soft tissues [16]. We found that through RANKL-RANK signaling a number of transcription factors and target genes were regulated in coordination, resulting in an alteration of the fundamental cellular processes of cancer cells. Notably, we found that RANKL-RANK signaling promotes the expression of RANKL, RANK, and c-Met through increased expression of transcription factors c-Myc/Max [16]. In concert with the activation of RANKL-RANK signaling, we also detected increased expression of VEGF in response to elevated HIF-1α transcription factors [6]. VEGF is a critical pro-angiogenic factor that induces proliferation and migration of endothelial cells within tumor vasculature [17]. Aberrant expression of VEGF and its receptors is associated with poor prognosis manifested by increased tumor vascularity, chemoresistance, local tumor invasion and distant metastases [18]. Elevated HIF-1α binds to the hypoxia-response elements (HREs) and activates VEGF promoter [19]. Neuropilin-1 (NRP-1), a VEGF co-receptor, was originally identified as a receptor for semaphorin 3, mediating neuronal guidance and axonal growth [20], that binds specifically VEGF165 but not VEGF121 on the cell surface of endothelial and tumor cells [20], [21]. NRP-1 lacks a typical kinase domain, and primarily functions as a co-receptor to form ligand-specific complexes. Aberrant upregulation of NRP-1 has been observed in high Gleason grade and metastatic PC and other solid tumors [22], [23]. Our lab reported that VEGF regulated an anti-apoptotic Mcl-1 gene through NRP1-dependent phosphorylation of c-Met in PC cells and broadened the function of this protein in cell signaling network [6].

Racial and ethnic differences in PC have been widely reported [24], [25]. While limited published data suggest potential differences in selective gene expression between aggressive versus indolent PC, data describing interracial comparisons of gene expression between the prostate glands from African-Americans and Caucasian-Americans are sparse. Kwabi-Addo and colleagues [26] reported differences in the specific promoter methylation of genes such as GSTPi, AR, RAR beta2, SPARC, TIMP3, and NKX2-5 in which higher methylation was found in African-Americans than in Caucasian-Americans. Using an immunohistochemical staining approach to profile PC specimens obtained from Caucasian-Americans, African-Americans, Chinese and Japanese, we found remarkable differences between these interracial groups with respect to their staining profiles of tumor suppressors, angiogenic and neuroendocrine factors [27], [28]. In the present study, we focus our attention on comparing RANKL-RANK signaling and its downstream effectors among Caucasian-Americans, African-Americans, and Chinese because of the significance of this signaling pathway in conferring PC bone and soft tissue metastases [5], [6], [8], [12]. We analyzed the levels of gene expression at a single cell level in clinical specimens obtained from these interracial groups using an established multiplexed quantum dot labeling (mQDL) technique to sequentially label each of the six signaling intermediates, capture multiple images, unmix and quantify the signals at the sub-cellular level and subject the data to a series of logistic statistical analyses to determine their predicting significance either alone or in combination with the clinical Gleason scores. Results of this study demonstrated that different downstream effectors of the RANKL-RANK signaling pathway can predict PC overall survival in interracial groups with PC.

## Materials and Methods

### Tissue specimens

This research did not involve any human participants. All the tissue specimens used in this study were archived formalin-fixed paraffin-embedded (FFPE) prostate cancer tissues. The use of specimens in research was approved by the institution review board of the Cedars-Sinai Medical Center (IRB # Pro21228). A total of 54 surgically removed FFPE primary prostate cancer specimens were obtained from patients from the Department of Pathology, the University of Virginia, Charlottesville, Virginia, and the Department of Pathology, Jilin University, Changchun, China, with documented cancer-caused death or survival information. Of the 54 specimens, 20 each were from Caucasian- and African- Americans and 14 from Chinese men. The number of patients, events, and mean (ranges) survival in months are: Caucasian-Americans-20, 16, and 74.6 (range 1–190); African- Americans-20, 18, and 46.3 (range 2–181); Chinese-14, 13 31.9 (range 1–107). The surgical procedures from which the tissue specimens were obtained were: Caucasian-Americans: 15 cases from transurethral resection of the prostate (TURP), 4 cases from radical prostatectomy (RP) and 1 case from needle biopsy (NBx); African-Americans: 18 cases from TURP and 2 cases from RP; Chinese: 1 case from TURP, 6 cases from suprapubic prostatectomy and 7 cases from needle biopsy. Efforts were made to ensure the consistency of Gleason grading; the histopathologic pattern of the specimens from the U. S. and China were scored by pathologists Dr. L. S. Zhao and Dr. Hua Yang from Jilin University during their visits at UTMDACC in Houston, TX and the University of Virginia, respectively, and confirmed by Dr. Henry F. Frierson, a genitourinary pathologist from the University of Virginia.

### Immunoassay reagents

The primary antibodies (Abs) and their sources were: mouse monoclonal Abs against HIF-1α (NB100–105) and RANKL (12A668) from Novus Biologicals (St. Charles, MO); rabbit polyclonal Abs to p-NFκB p65 or p-p65 (Ser 536), VEGF (A-20), and neuropilin-1 or NRP-1 (H286) from Santa Cruz Biotechnology, Inc. (Santa Cruz, CA); and rabbit polyclonal Ab to p-c-Met (pYpYpY1230/1234/1235) from Invitrogen (Carlsbad, CA). Secondary Abs used in the study were prepared in a cocktail of biotinylated Abs to mouse, rabbit, and goat IgG from Vector Laboratories Inc. (Burlingame, CA). Phosphate-buffered saline (PBS) and streptavidin-conjugated quantum dots (QD) at 565-, 585-, 605-, 625-, 655- and 705 nm wavelengths as 1 µM stock solution were from Invitrogen.

### Multiplexed QD labeling (mQDL)

We developed a mQDL protocol using streptavidin-coated QDs conjugated to biotinylated secondary Ab [6]. The experimental labeling protocol involved conjugating the primary Ab to a biotinylated secondary Ab, which in turn reacts with streptavidin-conjugated QD at a specified wavelength. This labeling procedure was repeated for multiple primary Abs against different biomarker antigens after optimization. The QD-labeled images were examined and captured under a Nuance multispectral camera and the cellular segmentation and quantification were performed by inForm software (Perkin Elmer; Waltham, MA). The multispectral QD image cube was further unmixed to its component images with distinct peak QD wavelengths. After removing the autofluorescence, the individual QD-labeled proteins can be detected.

The immunoreaction sequences and the dilutions of primary Ab and its pairing streptavidin-conjugated QD were: 1) anti-HIF-1α Ab (1∶40) and streptavidin-QD565 (1∶100); 2) anti-p-NFκB p65 Ab (1∶100) and streptavidin-QD585 (1∶100); 3) anti-VEGF Ab (1∶40) and streptavidin-QD605 (1∶100); 4) anti-neuropilin-1 Ab (1∶200) and streptavidin-QD625 (1∶100); 5) anti- p-c-Met Ab (1∶120) and streptavidin-QD655 (1∶100); 6) anti- RANKL Ab (1∶100) and streptavidin-QD705 (1∶100). All the primary Abs were incubated at 4^°^C, overnight and streptavidin-QD's reacted at 37°C, 1 hour. After 4 rinses with PBS-Triton (0.4%), the specimens were stained with DAPI and mounted. For negative control, primary Abs were replaced with isotype- and species-matched control Abs and applied to the immediate adjacent tissue sections from 5 pairs of tissue specimens from the studied cases. mQDL was performed in parallel with the tissue slide labeled with the testing primary Abs. The average cell-based intensity from the negative controls was subtracted from the test Ab labeling.

### Image capturing and biomarker expression intensity quantification

Multiplexed spectral imaging analyses including image acquisition and deconvolution using Nuance 3.0 software and signal quantification using inForm 1.3 software were performed as described in our previous report [6].

### Data description and statistical analyses

#### Data description

The primary outcome is defined as overall survival. Variables measured were Gleason score, race (Caucasian-Americans, African-Americans, and Chinese), and cell-based biomarker expression intensity in cytoplasm, C; nucleus, N; and cytoplasm plus nucleus, C+N of HIF-1α, p-p65, VEGF, NRP-1, p-c-Met and RANKL. Biomarker measurements for each patient were averaged from 4–5 images captured from each of the tumor tissue sites on the slide. An average of 27 images/tissue slide was taken (a range of 4–114 images) which break down to: Caucasian-Americans, 5–114 images; African-Americans, 5–57 images; Chinese, 4–46 images. The total sample size of this study is 54 patients (Caucasian-American, N = 20; African-American, N = 20; Chinese, N = 14). The averaged numbers of cells analyzed with minimum to maximum and standard deviation (SD) are: Caucasian-Americans, 17,207 (1,363–50,488, SD = 14,702); African-Americans, 12,541 (2,062–24,612; SD = 6,899); Chinese, 3,290 (794–14,770, SD = 3,531).

#### Statistical analysis

The Kaplan and Meier method was used to estimate overall survival and the logrank test to compare groups. Multivariable proportional hazards regression using forward variable selection was used to assess which biomarkers are predictive of overall survival in the presence of covariates. Proportional hazards assumption was evaluated graphically and analytically, and martingale residuals were used to ensure that the models are appropriate. Critical significance level was set to 5%.

## Results

**Figure 1:**
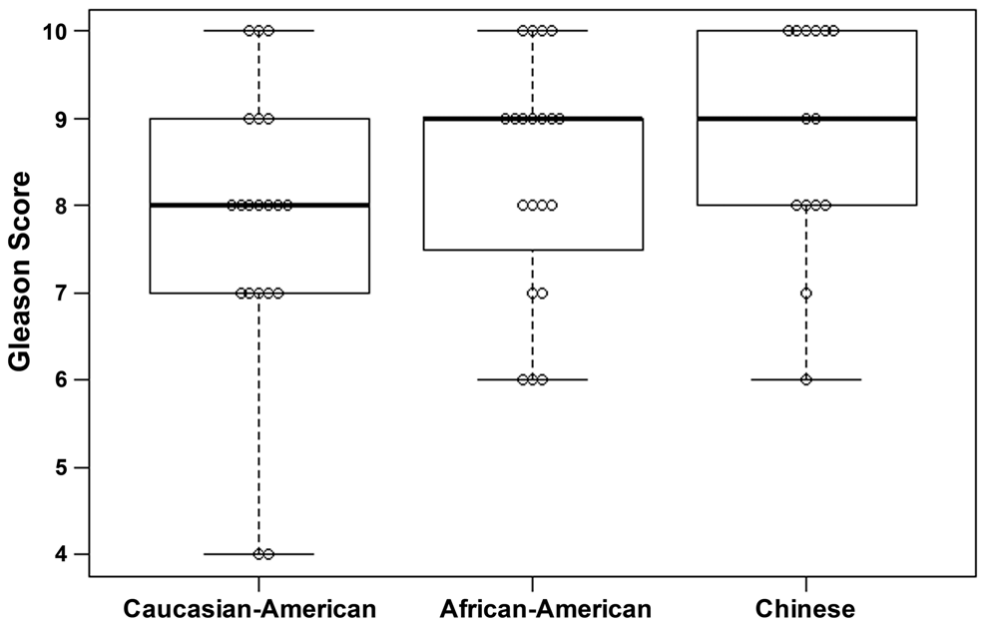
Gleason score box-plots by race.

**Figure 2:**
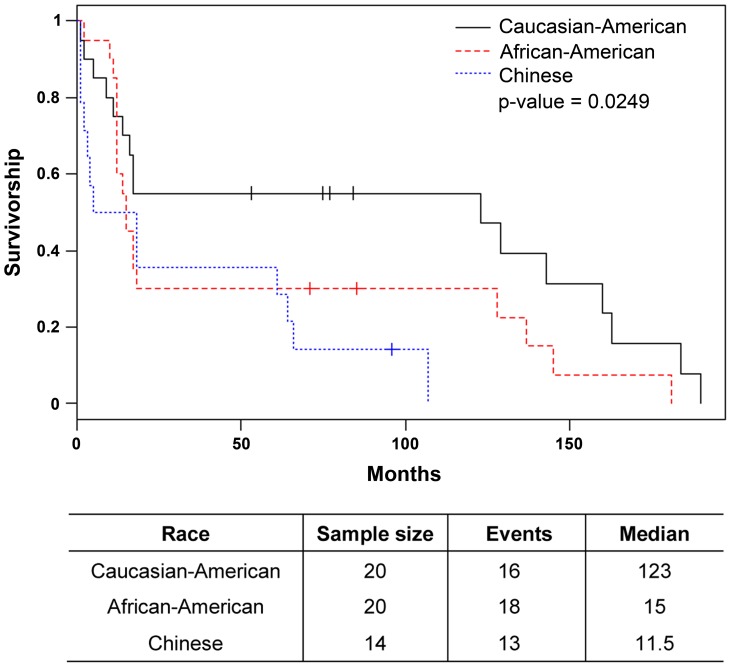
Log-rank test of overall survival by race including all cases (N = 54, number of events  = 47).

**Figure 3:**
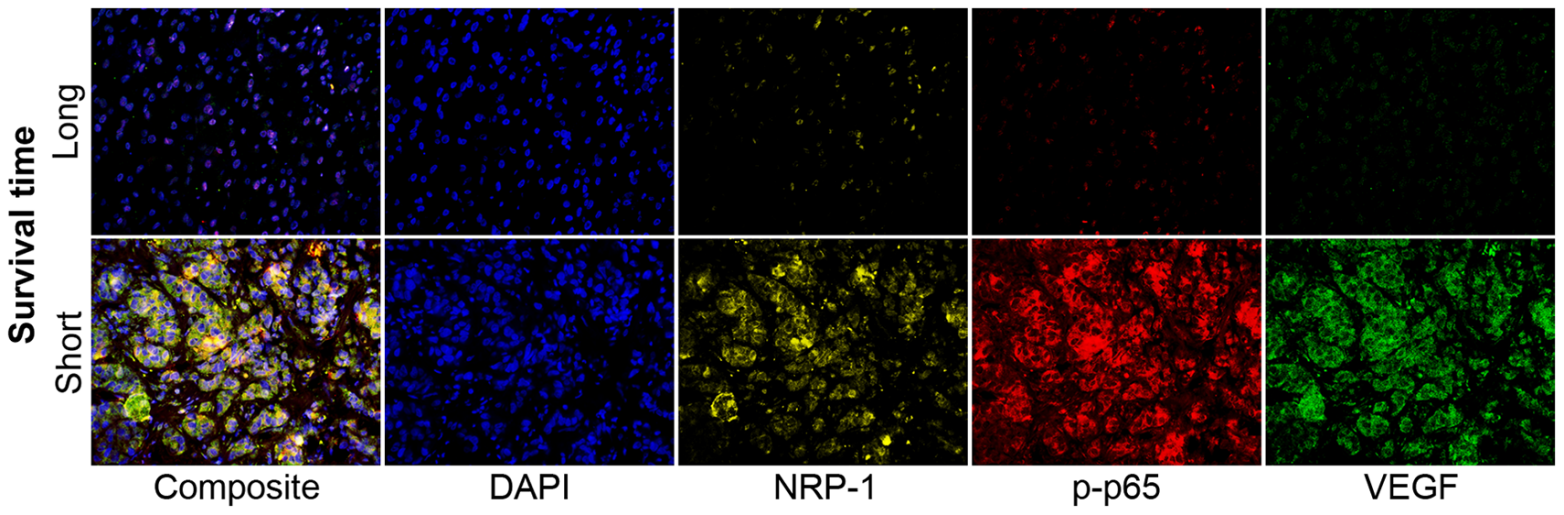
Unmixed NRP-1, p-p65 and VEGF protein expression images from the mQDL of tissues from a Chinese patient who survived for 66 months (top panels) vs a patient who survived for 2 months (bottom panels).

**Figure 4:**
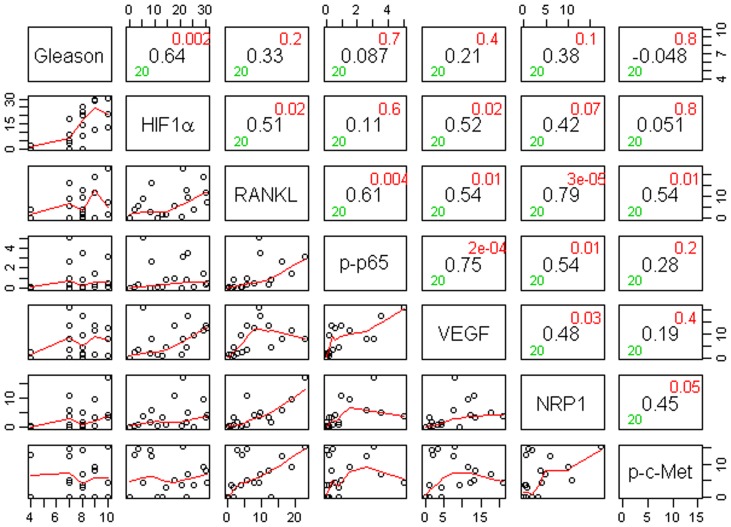
Correlogram with pairwise correlations between Gleason scores and biomarker expression in (cytoplasm+nucleus) for Caucasian-Americans (N = 20). The main diagonal has the covariate name. At the horizontal and vertical intersection of each covariate, Pearson correlation coefficient (center, in black), and the associated p-value (top-right corner, in red) are shown.

**Figure 5:**
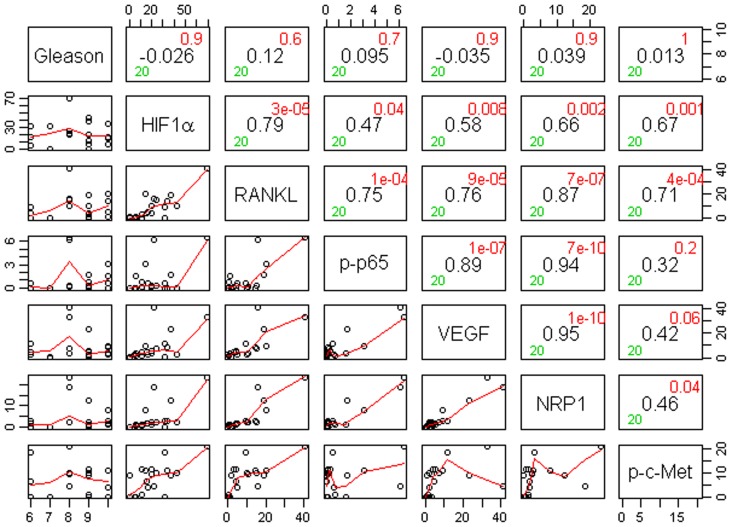
Correlogram with pairwise correlations between Gleason scores and biomarker expression in (cytoplasm+nucleus) for African-Americans (N = 20). The main diagonal has the covariate name. At the horizontal and vertical intersection of each covariate, Pearson correlation coefficient (center, in black), and the associated p-value (top-right corner, in red) are shown.

**Figure 6:**
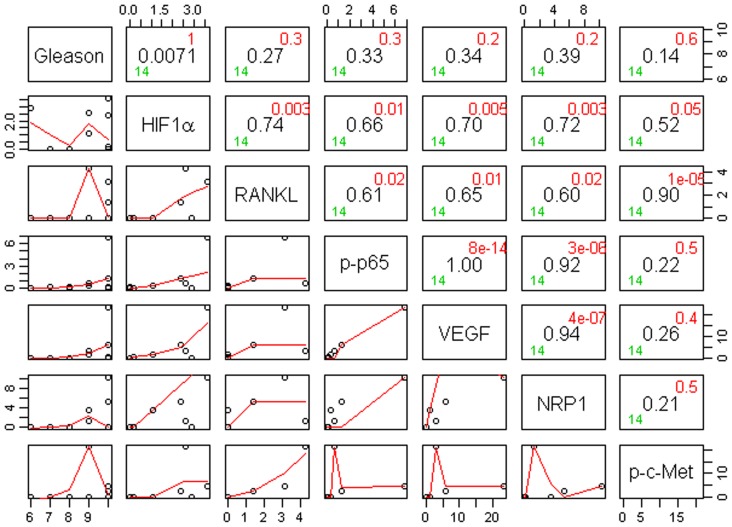
Correlogram with pairwise correlations between Gleason scores and biomarker expression in (cytoplasm+nucleus) for Chinese (N = 14). The main diagonal has the covariate name. At the horizontal and vertical intersection of each covariate, Pearson correlation coefficient (center, in black), and the associated p-value (top-right corner, in red) are shown.

**Figure 7:**
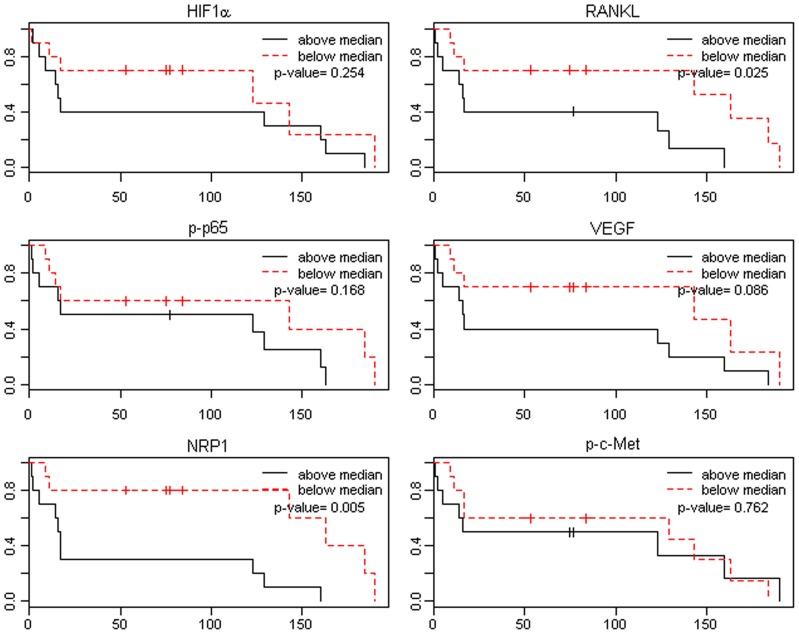
Overall survival by biomarker expression in (cytoplasm+nucleus) for Caucasian-Americans (N = 20, number of events  = 16). Log-rank test p-value is presented. X-axis is survival time in months. Y-axis is the proportion of surviving.

**Figure 8:**
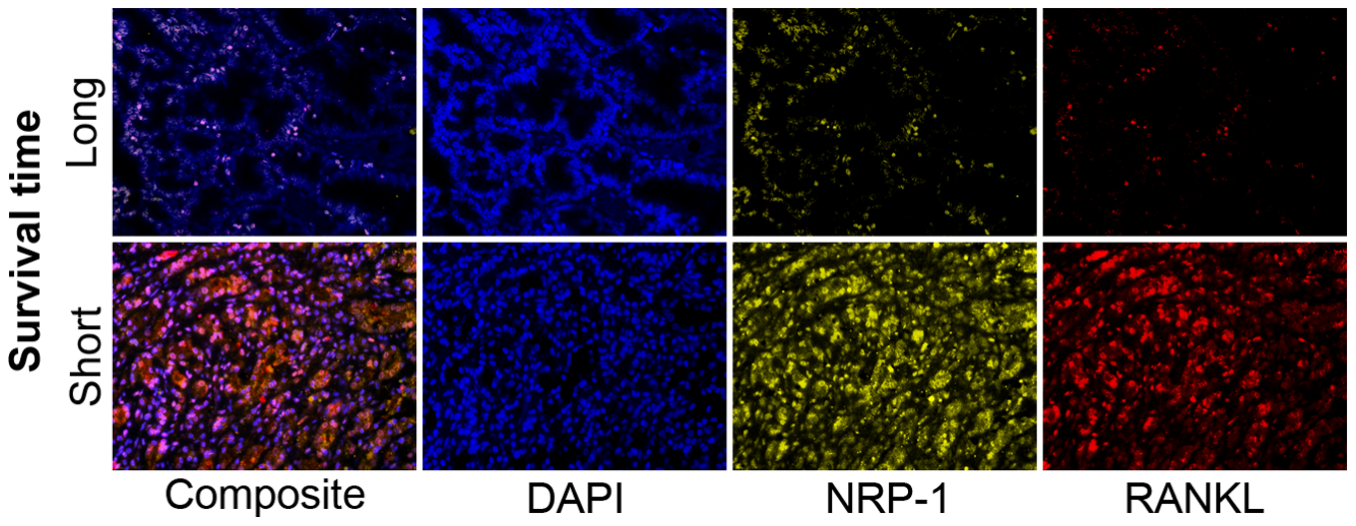
Unmixed mQDL images of NRP-1 and RANKL expression from representative tissues (one long survival, one short survival) from a Caucasian-American patient who survived for 163 months (top panels) and a patient who survived for 2 months (bottom panels).

**Figure 9:**
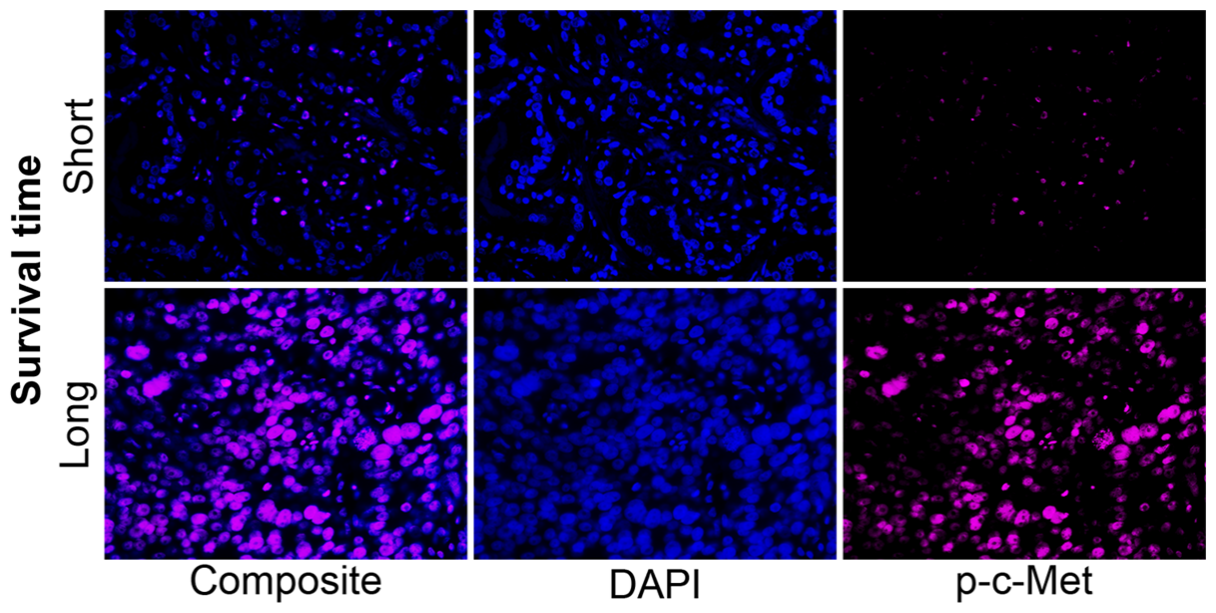
Unmixed mQDL image of p-c-Met protein expression in an African-American patient who survived for 85 months (top panels) vs an African-American patient who survived for 12 months (bottom panels).

**Figure 10:**
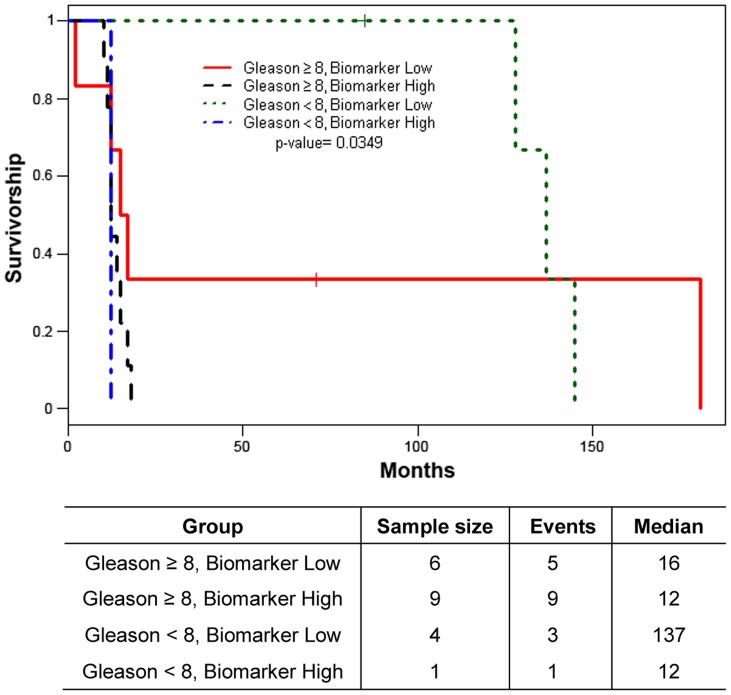
Overall survival with dummy variables for interaction between binary nuclear p-c-Met biomarker and binary Gleason score for African-Americans. (N = 20, number of events  = 18). Log-rank test p-value is presented. ‘Biomarker High’ indicates biomarker values above the median of the (continuous) biomarker. ‘Biomarker Low’ indicates biomarker values below or equal to the median of the (continuous) biomarker.

**Figure 11:**
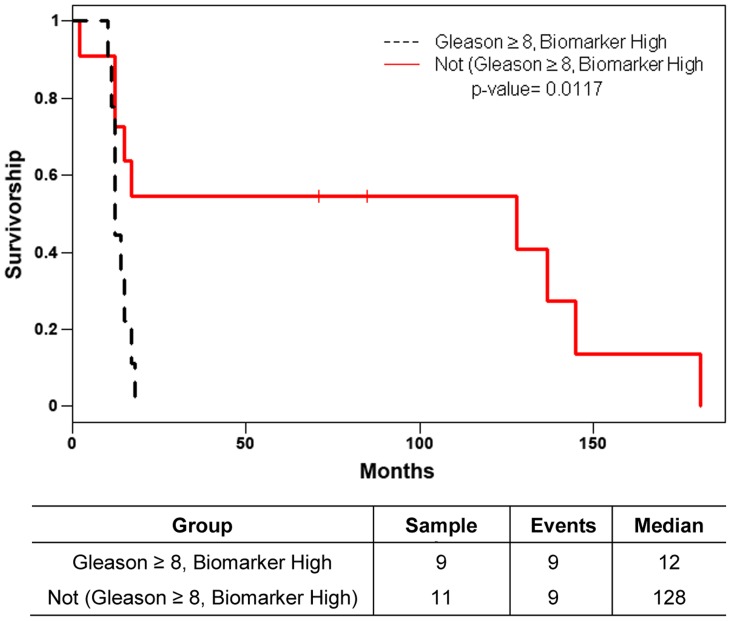
Overall survival with dummy variable for Gleason ≥8, nuclear p-c-Met Biomarker High, African American (N = 20, number of events  = 18). Log-rank test *p*-value is presented. ‘Biomarker High’ indicates biomarker values above the median of the (continuous) biomarker. ‘Biomarker Low’ indicates biomarker values below or equal to the median of the (continuous) biomarker.

Gleason score box-plots by race among the 3 studied patient groups showed clustered high Gleason scores in Caucasian-Americans, African-Americans and Chinese PC patients (Figure 1). Figure 2 shows a significant difference in overall survival by race including all cases (N = 54, number of events  = 47) by Log-rank test (*p* = 0.0249). Furthermore, there was significant difference in biomarkers' mean and standard deviations for combined sample and by each race (Table 1) where Chinese differ from both Caucasian-Americans and African-Americans. To identify potential biomarkers that predict overall survival of patients with PC, we then analyzed all data independently for each race. Continuous analyses by univariate proportional hazard regression models with Gleason score and biomarkers for Caucasian-American patients (Table 2) showed that RANKL and NRP-1 expression in cytoplasm (C) plus nucleus (N) were significantly correlated with the overall survival of patients with PC, *p*-value  = 0.0053 and 0.0029, respectively. Significant associations were also found when expression in C or N was analyzed separately. Similar analyses showed that NRP-1 in C, N, and C+N; p-p65 in C, C+N; and VEGF in C were significantly correlated with the overall survival of Chinese patients with PC (Table 3). Figure 3 shows NRP-1, p-p65 and VEGF protein expression images from the mQDL of tissues obtained from a Chinese patient who survived for 66 months (top panels) vs a patient who survived for 2 months (bottom panels). In contrast, with the exception of Gleason score (*p* = 0.027), none of the 6 biomarkers reached significant association with survival time of African-American patients analyzed by the same method (Table 4). Correlograms (Figures 4, 5, 6) showed pair-wise correlations between biomarkers with each other, and biomarkers with Gleason scores among Caucasian-Americans, African-Americans and Chinese patients, respectively. The main diagonal shows the covariate names for each pair-wise comparison. The center at the horizontal and vertical interaction of each covariate is the Pearson correlation coefficient and at the top right is the associated p value. Results showed that there were significant correlations between most of the biomarker pairs (in bold) irrespective of the race but only HIF-1α correlates with Gleason score for Caucasian-American patients, *p*-value  = 0.002. Figure 7 shows additional discretized visualizations of the effect of categorized biomarkers on overall survival of the Caucasian patients as analyzed by Kaplan and Meier method and log-rank test to compare biomarker protein expression in cytoplasm plus nucleus categorized in two groups, high and low, using the median as a cutoff point. RANKL and NRP-1 correlated significantly with overall survival, with *p*-value  = 0.025 and 0.005, respectively. Figure 8 presents unmixed mQDL images of NRP-1 and RANKL expression from representative tissues from a Caucasian-American patient who survived for 163 months (top panels) *vs.* a patient who survived only 2 months (bottom panels). Similar analyses performed in African-American and Chinese patients did not show a correlation between RANKL and NRP-1 biomarkers and patient overall survival (data not included). For African-Americans, although only Gleason scores were significant in the univariate model (Table 4), nuclear p-c-Met became a significant predictor in combination with Gleason score (Table 5) in a multivariable proportional hazard regression model (*p* = 0.025 and *p* = 0.044, respectively). Figure 9 shows the unmixed mQDL images of p-c-Met protein expression in an African-American patient who survived for 85 months (top panels) vs an African-American patient who survived for 12 months (bottom panels). To visualize the effect of these two variables on overall survival, Gleason score was categorized into two groups: ≥8 and <8, and nuclear p-c-Met was categorized in two groups, high and low, using the median as a cutoff point (Figure 10, *p* = 0.0349). To further explore in a systematic way whether combining Gleason score and a biomarker may improve the prediction of overall survival, all biomarkers that were significant predictors of overall survival in univariate models from Tables 2, 3, 4 were categorized in two groups, high and low, using the median as a cutoff point. Gleason score was categorized into two groups: ≥8 and <8 and the two dichotomous variables were combined to generate four groups: Gleason ≥8 and Biomarker High, Gleason ≥8 and Biomarker Low, Gleason <8 and Biomarker High, and Gleason <8 and Biomarker Low. Three dummy variables were created, using the group Gleason <8 and Biomarker Low as the reference, and multivariable proportional hazards regression using forward variable selection was used to select the best model to predict overall survival. The results of these multivariable models are shown in Table 6. While there was no categorized Gleason/Biomarker group predictive of overall survival for Caucasian-Americans and Chinese, for African-Americans the results with the categorized groups agreed with the multivariable continuous predictor model and details of the analysis as shown in Table 7. The final model is shown in Table 8 and displayed in Figure 11 where only “Gleason ≥8/Biomarker High” is a significant predictor of overall survival in African American patients with prostate cancer (*p* = 0.0117).

**Table 1:** Biomarker summary statistics for combined sample and by race.

Biomarker	All (n = 54)	Caucasian-American (n = 20)	African-American (n = 20)	Chinese (n = 14)
HIF1-α (C+N)	13.23±14.24	14.24±10.55	20.84±16.70	0.91±1.34
RANKL (C+N)	5.89±8.04	6.80±6.80	8.67±10.17	0.63±1.38
p-p65 (C+N)	0.95±1.71	1.00±1.43	1.08±1.94	0.67±1.80
VEGF (C+N)	6.14±8.61	6.63±6.34	8.28±11.18	2.40±6.20
NRP-1 (C+N)	3.24±5.10	3.51±4.55	4.20±6.50	1.47±3.03
p-c-met (C+N)	5.42±6.08	5.93±5.58	7.28±6.10	2.04±5.71
HIF1-α (N)	8.15±8.86	8.62±6.39	12.98±10.52	0.60±0.94
RANKL (N)	3.88±5.00	4.31±4.05	5.85±6.31	0.46±1.00
p-p65 (N)	0.44±0.81	0.47±0.70	0.51±0.96	0.29±0.77
VEGF (N)	4.04±5.38	4.23±3.83	5.68±7.07	1.42±3.43
NRP-1 (N)	1.91±2.90	2.03±2.53	2.63±3.77	0.71±1.40
p-c-Met (N)	3.15±3.46	3.28±2.83	4.48±3.79	1.07±2.96
HIF1-α (C)	5.09±5.65	5.64±4.62	7.87±6.50	0.31±0.54
RANKL (C)	2.03±3.12	2.50±2.84	2.86±3.94	0.18±0.38
p-p65 (C)	0.52±0.91	0.54±0.74	0.58±1.00	0.39±1.03
VEGF (C)	2.11±3.40	2.40±2.69	2.60±4.29	0.99±2.79
NRP-1 (C)	1.35±2.24	1.51±2.04	1.60±2.77	0.76±1.63
p-c-met (C)	2.30±2.73	2.67±2.84	2.86±2.40	0.98±2.75

Values in Mean ± standard deviation. C =  Cytoplasm; N =  Nucleus.

**Table 2:** Univariate proportional hazard regression models with Gleason score and biomarkers for Caucasian-Americans (N = 20, number of events  = 16).

Covariate	Coefficient	Hazard ratio	*p*-value
Gleason score	0.468	1.6	0.075
HIF1-α (C+N)	0.0436	1.04	0.14
**RANKL (C+N)**	**0.146**	**1.16**	**0.0053**
p-p65 (C+N)	0.197	1.22	0.21
VEGF (C+N)	0.0441	1.05	0.25
**NRP-1 (C+N)**	**0.197**	**1.22**	**0.0029**
p-c-met (C+N)	−0.0218	0.978	0.67
HIF1-α (N)	0.085	1.09	0.091
**RANKL (N)**	**0.247**	**1.28**	**0.0072**
p-p65 (N)	0.349	1.42	0.27
VEGF (N)	0.0809	1.08	0.21
**NRP-1 (N)**	**0.346**	**1.41**	**0.0039**
p-c-Met (N)	−0.0337	0.967	0.74
HIF1-α (C)	0.0593	1.06	0.3
**RANKL (C)**	**0.299**	**1.35**	**0.0058**
p-p65 (C)	0.436	1.55	0.16
VEGF (C)	0.0826	1.09	0.34
**NRP-1 (C)**	**0.446**	**1.56**	**0.0024**
p-c-met (C)	−0.0513	0.95	0.62

C =  Cytoplasm; N =  Nucleus.

**Table 3:** Univariate proportional hazard regression models with patient Gleason score and biomarkers for Chinese (N = 14, number of events  = 13).

Covariate	Coefficient	Hazard ratio	*p*-value
Gleason score	0.45	1.58	0.076
HIF1-α (C+N)	−0.0788	0.924	0.74
RANKL (C+N)	0.0589	1.06	0.76
**p-p65 (C+N)**	**0.362**	**1.44**	**0.049**
VEGF (C+N)	0.104	1.11	0.054
**NRP-1 (C+N)**	**0.248**	**1.28**	**0.033**
p-c-met (C+N)	−0.00726	0.993	0.87
HIF1-α (N)	0.0982	1.1	0.78
RANKL (N)	0.0758	1.08	0.77
p-p65 (N)	0.823	2.28	0.056
VEGF (N)	0.182	1.2	0.067
**NRP-1 (N)**	**0.51**	**1.67**	**0.042**
p-c-Met (N)	−0.0136	0.987	0.88
HIF1-α (C)	−0.587	0.556	0.35
RANKL (C)	0.255	1.29	0.72
**p-p65 (C)**	**0.648**	**1.91**	**0.044**
**VEGF (C)**	**0.238**	**1.27**	**0.046**
**NRP-1 (C)**	**0.47**	**1.6**	**0.028**
p-c-met (C)	−0.0156	0.985	0.87

C =  Cytoplasm; N =  Nucleus.

**Table 4:** Univariate proportional hazard regression models with patient Gleason score and biomarkers for African-Americans (N = 20, number of events  = 18).

Covariate	Coefficient	Hazard ratio	*p*-value
Gleason score	0.534	1.71	0.027
HIF1-α (C+N)	0.0131	1.01	0.3
RANKL (C+N)	0.0124	1.01	0.56
p-p65 (C+N)	−0.102	0.903	0.42
VEGF (C+N)	−0.0158	0.984	0.44
NRP-1 (C+N)	−0.0204	0.98	0.58
p-c-met (C+N)	0.0658	1.07	0.091
HIF1-α (N)	0.0202	1.02	0.31
RANKL (N)	0.0239	1.02	0.49
p-p65 (N)	−0.25	0.779	0.34
VEGF (N)	−0.0167	0.983	0.6
NRP-1 (N)	−0.0226	0.978	0.72
p-c-Met (N)	0.116	1.12	0.06
HIF1-α (C)	0.0336	1.03	0.31
RANKL (C)	0.0227	1.02	0.69
p-p65 (C)	−0.155	0.856	0.52
VEGF (C)	−0.0639	0.938	0.28
NRP-1 (C)	−0.0686	0.934	0.43
p-c-met (C)	0.144	1.15	0.15

C =  Cytoplasm; N =  Nucleus.

**Table 5:** Multivariable proportional hazard regression models with patient Gleason score, nuclear p-c-met, after variable selection, for African-Americans (N = 20, number of events  = 18).

Covariate	Coefficient	Hazard ratio	*p*-value	Null martingale residual analysis (*p*-value)
Gleason score	0.611	1.84	0.025	0.129
Nuclear p-c-met (continuous)	0.139	1.15	0.044	0.445

**Table 6:** Multivariable proportional hazards regression using forward variable selection was used to select the best model to predict overall survival.

Population	Biomarker	Categorized Gleason/Biomarker group Predictive of overall survival
Caucasian-American	RANKL (C+N)	none
Caucasian-American	NRP-1 (C+N)	none
Caucasian-American	RANKL (N)	none
Caucasian-American	NRP-1 (N)	none
Caucasian-American	RANKL (C)	none
Caucasian-American	NRP-1 (C)	none
African-American	p-c-met (C+N)	none
African-American	p-c-Met (N)	**[Gleason ≥8, Biomarker** **High]**
Chinese	p-p65 (C+N)	None
Chinese	NRP-1 (C+N)	None
Chinese	NRP-1 (N)	none
Chinese	p-p65 (C)	none
Chinese	VEGF (C)	none
Chinese	NRP-1 (C)	none

C =  Cytoplasm; N =  Nucleus.

All biomarkers that were significant predictors of overall survival in univariate models from Tables 2, 3, 4 were categorized in two groups, high and low, using the median as a cutoff point. Gleason score was categorized into two groups: ≥8 and <8 and the two dichotomous variables were combined to generate four groups: Gleason ≥8 and Biomarker High, Gleason ≥8 and Biomarker Low, Gleason <8 and Biomarker High, and Gleason <8 and Biomarker Low. ‘Biomarker High’ indicates biomarker values greater than the median of the (continuous) biomarker.

**Table 7:** Multivariable proportional hazard regression models with all binary dummy variables for African-American (N = 20, number of events  = 18).

Binary dummy variable	Coefficient	Hazard ratio	*p*-value	Null martingale residual analysis (*p*-value)
Gleason ≥8, Biomarker High	**1.924**	**6.85**	**0.015**	**0.5**
Gleason ≥8, Biomarker Low	0.622	1.86	0.42	0.06
Gleason <8, Biomarker High	2.49	12.06	0.052	0.71

‘Biomarker High’ indicates biomarker values above the median of the (continuous) biomarker. ‘Biomarker Low’ indicates biomarker values below or equal to the median of the (continuous) biomarker.

**Table 8:** Multivariable proportional hazard regression model, with significant binary dummy variable for African-Americans (N = 20, number of events  = 18).

Binary dummy variable	Coefficient	Hazard ratio	*p*-value	Null martingale residual analysis (*p*-value)
Gleason ≥8, Biomarker High	**1.34**	**3.83**	**0.019**	**0.395**

‘Biomarker High’ indicates biomarker values above the median of the (continuous) biomarker.

## Discussion

Increasing evidence suggests that improved prostate cancer prognosis using biomarkers differentially expressed in tissues, cells and body fluids among patients with either indolent or aggressive disease could reduce healthcare costs and patient anxiety and suffering, and improve the overall effectiveness of the treatment plan. While histopathology and immunohistochemistry have provided the “gold standards” for PC diagnosis at the cellular level, the quantitative and prognostic aspects of these techniques have not been critically evaluated. In this communication, we used a multiplexed quantum-dot labeling based quantitative histopathology approach at a single cell level as reported previously by our group [6] to assess the expression of cell signaling pathway components downstream from a RANK- and c-Met-mediated signaling network in clinical PC specimens collected from interracial groups, comprised of Caucasian-Americans, African-Americans and Chinese patients, and assessed if these signaling pathway components can predict the survival of PC patients. Activation of RANK- and c-Met-mediated signaling by tumor- and host-derived RANKL has been shown to drive cancer bone and soft tissue metastases in human prostate, breast, lung, kidney and liver cancers. Upon activation of these signaling pathways, we noted increased expression of HIF-1α, VEGF, NRP-1, RANKL, c-Met, and phosphorylated c-Met in cells that conferred resistance to castration and development of a metastatic phenotype in a human PC xenograft model [6]. We found the following interracial differences in the activation of RANK- and c-Met-mediated downstream cell signaling networks in PC cells. 1) RANKL and NRP-1 expression predicts survival of Caucasian-Americans with PC (Figure 7). 2) In African-Americans, combined Gleason score ≥8 and nuclear p-c-Met expression predicts survival (Figures 10 &11). 3) NRP-1, p-NF-κB p65 and VEGF are predictors for overall survival in Chinese men with PC (Table 3). 4) Despite differences in the prediction of overall survival of PC patients by different signaling pathway components, all racial groups shared the common downstream signaling components following activation of RANK- and c-Met-mediated signaling. This is revealed by the highly significant pairwise correlation among these signaling components plotted by the Correlogram (Figures 4, 5, 6) with pair-wise correlations in all three racial groups. Although at the present time there is no scientific explanation for why different signaling components predict survival in three distinct racial groups of PC patients, we speculate that the regulatory elements, including quantitative aspects of receptors, ligands, and interactions among effector molecules, controlling overall RANK- and c-Met-mediated downstream signaling could be different among interracial groups. These results, however, collectively support interracial differences of RANK- and c-Met-mediated cell signaling network which governs the survival of PC patients.

The present study is the first to use cell-based multispectral quantum dot labeling of rational pathway-associated biomarkers coupled with detailed statistical analyses to test their predicting capability for overall survival of patients with prostate cancer. To reduce the potential variables introduced by tissue specimen processing we chose to use specimens from the same hospital for each racial group. Despite the limited number of patient specimens available for the study, the newly established multiplexed quantum dot labeling and quantification technology demonstrated the predictive utility of RANK- and c-Met-mediated convergent signaling pathways for predicting the overall survival of patients with PC. Our results demonstrated that among the interracial groups, different sets of biomarkers could be used as predictors for survival. Our findings further support the well documented epidemiological disparities among Caucasian-American, African-American and Chinese patients with PC. We will further expand the sample size in a retrospective validation study and advance to prospective studies of disease progression and patient survival, employing circulating tumor cells (CTC) from patients undergoing treatments to validate our findings and discover other new biomarkers for prostate cancer diagnosis and prognosis to assist with clinical decision-making.
